# Finerenone combined with guideline-directed medical therapy in patients with post-myocardial infarction heart failure: a real-world study

**DOI:** 10.3389/fphar.2026.1754115

**Published:** 2026-05-29

**Authors:** Di Zhao, Jingsong Xia, Zhenhua Yang, Yonghong Yong, Liansheng Wang, Jiabao Liu

**Affiliations:** 1 Department of Cardiology, The First Affiliated Hospital with Nanjing Medical University, Nanjing, China; 2 The Second Clinical Medical College, Nanjing Medical University, Nanjing, China

**Keywords:** cardiac remodeling, finerenone, guideline-directed medical therapy, post-myocardial infarction heart failure, real-world study

## Abstract

**Objective:**

To evaluate the short-term impact of finerenone combined with guideline-directed medical therapy (GDMT) on cardiac structure, function, and renal parameters in Chinese patients with post-myocardial infarction heart failure in a real-world setting.

**Methods:**

In this prospective observational cohort study, 67 patients received finerenone plus optimized GDMT for 3 months. Changes were assessed using echocardiography (measuring LVEF, GLS, left atrial strain and volume index), laboratory tests (NT-proBNP, serum potassium, renal function, urinary albumin-to-creatinine ratio [UACR]), and functional assessments (6-min walk test, Minnesota Living with Heart Failure Questionnaire).

**Results:**

Treatment significantly improved cardiac function and structure. LVEF increased from 49 (2)% to 53 (2)% (P < 0.001), GLS improved from 14 (1)% to 16 (1)% (P < 0.001), and left atrial reservoir strain increased from 27 (2)% to 32 (2)% (P < 0.001). Structural reverse remodeling was evidenced by reduced left ventricular end-diastolic diameter (54.85 [1.08] to 52.85 [0.96] mm, P < 0.001) and left atrial volume index (38.78 [2.69] to 36.25 [2.61] mL/m^2^, P < 0.001). Concurrently, a significant reduction in UACR was observed from 3.41 ± 1.52 mg/g to 2.76 ± 0.90 mg/g (P < 0.001). Clinically, NT-proBNP decreased from 1,249 ± 1,251 to 431 ± 319 pg/mL (P < 0.001), 6-min walk distance increased by 41.11 m (P < 0.001), and quality of life scores improved (a reduction of 11.59 points, P < 0.001). Serum potassium and renal function (serum creatinine) remained stable.

**Conclusion:**

In this real-world cohort of 67 post-myocardial infarction heart failure patients, adding finerenone to GDMT for 3 months was associated with significant improvements in cardiac structure and function, a reduction in UACR, and better functional capacity and quality of life. Nevertheless, due to the single-arm design, these findings are exploratory and hypothesis-generating.

## 1 Introduction

Heart failure represents a common terminal stage of various cardiovascular diseases, with its global prevalence continuously rising, posing a substantial public health burden (Heidenreich et al., 2022). Although significant progress has been made in the pharmacological treatment of heart failure in recent years—particularly for patients with heart failure with reduced ejection fraction (HFrEF) (Murphy et al., 2020), where guideline-directed medical therapy (GDMT) centered around angiotensin receptor-neprilysin inhibitors (ARNI), beta-blockers (BB), mineralocorticoid receptor antagonists (MRA), and sodium-glucose cotransporter-2 inhibitors (SGLT2i) has markedly improved outcomes (McDonagh et al., 2023; Zhao et al., 2024)—clinical management for patients with heart failure with preserved ejection fraction (HFpEF) and mildly reduced ejection fraction (HFmrEF) remains challenging (Writing Committee and Members, 2022), with relatively limited therapeutic options and ongoing difficulties in improving clinical prognosis.

Overactivation of the mineralocorticoid receptor plays a pivotal role in the pathophysiology of heart failure, primarily by mediating myocardial fibrosis, inflammatory responses, and promoting adverse ventricular remodeling, thereby exacerbating the progressive deterioration of cardiac function (El Mouhayyar et al., 2025). Finerenone, a novel non-steroidal mineralocorticoid receptor antagonist, has garnered significant attention due to its higher receptor selectivity, stronger affinity, and improved safety profile. Its cardiorenal protective effects have been validated in several large-scale clinical trials (Vaduganathan et al., 2024; Solomon et al., 2024a). The phase III FINEARTS-HF trial demonstrated that in symptomatic heart failure patients with a left ventricular ejection fraction (LVEF) ≥40%, finerenone significantly reduced the risk of cardiovascular death and total heart failure events by 16%, with benefits observed as early as 28 days after initiation (Solomon et al., 2024b). Concurrently, robust evidence from studies such as FIDELIO-DKD (Agarwal et al., 2022) has established that finerenone also provides clear benefits in reducing cardiovascular events and improving renal outcomes in patients with chronic kidney disease and type 2 diabetes, further solidifying its foundational role in cardiorenal protection.

Acute myocardial infarction is one of the most common and significant etiologies of heart failure (Jenca et al., 2021). In patients with post-myocardial infarction heart failure, early and comprehensive intervention to mitigate ventricular remodeling, reverse abnormal cardiac structure, and improve cardiac function is crucial for enhancing long-term prognosis. Currently, real-world clinical data on the efficacy and safety of the novel combination therapy comprising finerenone and GDMT (including ARNI, BB, SGLT2i and Vericiguat), particularly evidence specific to the Chinese population with post-myocardial infarction heart failure, remains relatively scarce.

Therefore, this study aims, through a prospective observational cohort design, to systematically investigate the short-term effects of finerenone combined with GDMT on cardiac structure and function, serum biomarkers, exercise tolerance, and quality of life in Chinese patients with post-acute myocardial infarction heart failure (encompassing the full LVEF spectrum including HFrEF, HFmrEF, and HFpEF). The findings are intended to provide reference evidence from a Chinese population to inform the broader application and optimization of this innovative treatment strategy in real-world clinical practice.

## 2 Methods

### 2.1 Study design and intervention

This study was a prospective, observational cohort study designed to investigate the short-term effects of finerenone combined with guideline-directed medical therapy (GDMT) on cardiac structure and function in Chinese patients with post-acute myocardial infarction heart failure in a real-world clinical setting.

**FIGURE 1:**
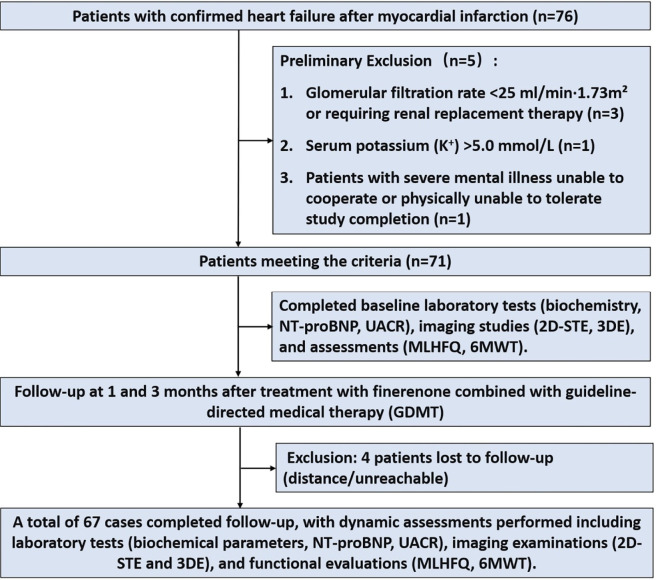
Study flow diagram.

The study subjects were consecutive outpatients or inpatients with post-myocardial infarction heart failure enrolled from the Department of Cardiology, the First Affiliated Hospital of Nanjing Medical University, between September 2024 and October 2025. After screening, 67 patients who met all inclusion criteria were ultimately included in the final analysis. Based on baseline left ventricular ejection fraction (LVEF), patients were categorized into a heart failure with preserved ejection fraction (HFpEF) group (n = 31) and a non-HFpEF group (n = 36), which included 27 patients with heart failure with mildly reduced ejection fraction (HFmrEF) and 9 patients with heart failure with reduced ejection fraction (HFrEF). The study flowchart is detailed in Figure 1.

The study protocol was reviewed and approved by the Ethics Committee of the First Affiliated Hospital of Nanjing Medical University (Approval No.: 2023-SR-646). All enrolled patients provided written informed consent before participation.

### 2.2 Inclusion criteria

Patients were required to meet all of the following criteria: (1) Age≥18 years, regardless of gender; (2) History of diagnosed myocardial infarction for more than 3 months; (3) New York Heart Association (NYHA) functional class II–IV; (4) Left ventricular ejection fraction (LVEF) < 55% as measured by echocardiography; (5) N-terminal pro-B-type natriuretic peptide (NT-proBNP) level≥300 pg/mL for patients in sinus rhythm, or NT-proBNP level≥900 pg/mL for patients with atrial fibrillation; (6) Estimated glomerular filtration rate (eGFR)≥25 mL/min/1.73 m^2^, calculated using the Chronic Kidney Disease Epidemiology Collaboration (CKD-EPI) formula; (7) Random serum potassium concentration ≤5.0 mmol/L; (8) Hemoglobin level≥90 g/L.

### 2.3 Exclusion criteria

Patients meeting any of the following criteria were excluded: (1) eGFR <25 mL/min/1.73 m^2^; (2) Random serum potassium concentration >5.0 mmol/L; (3) Presence of uncorrectable risk factors for hyperkalemia, such as current use of potassium-sparing diuretics (e.g., spironolactone, eplerenone); (4) Severe hepatic insufficiency, defined as Child-Pugh class B or higher; (5) Hospitalization for acute myocardial infarction, unstable angina, or acute heart failure within 3 months prior to enrollment; (6) Presence of severe uncontrolled arrhythmias, such as symptomatic ventricular tachycardia or ventricular fibrillation; (7) Moderate or severe valvular stenosis or regurgitation that has not been corrected by surgery or intervention; (8) Pregnancy or lactation; (9) Current or previous diagnosis of malignancy (except for basal cell carcinoma).

### 2.4 Sample size and post-hoc power analysis

The sample size was determined based on the expected change in left ventricular ejection fraction (LVEF) after 3 months of treatment. From our preliminary data (n = 32), the mean LVEF increase was 4.0%, with a conservative estimate of the standard deviation of the change set at 8.0% (reflecting typical variability in real world heart failure populations). For a paired t-test with a two-sided significance level of α = 0.05 and a desired power of 80%, the required sample size was calculated to be 50 patients. To account for a potential 20% dropout rate and to improve precision, we enrolled 67 patients.

After data collection, a post-hoc power analysis was performed for the primary endpoint (LVEF change). With the observed mean difference of 4.0% and a standard deviation of the paired difference of 7.8% (derived from the actual data of the 67 patients), the paired t-test achieved a power of 0.88 at α = 0.05 (two tailed). This indicates that the study was adequately powered to detect the prespecified effect size for the primary endpoint.

### 2.5 Trial registration statement

This study is a prospective observational cohort study, not a clinical trial as defined by the ICMJE (i.e., no prospective assignment of participants to intervention). Therefore, prospective registration in a public trial registry was not mandatory. Nevertheless, in the interest of transparency and in response to the reviewer’s suggestion, we acknowledge that the study was not pre-registered. The study protocol was reviewed and approved by the Ethics Committee of the First Affiliated Hospital of Nanjing Medical University (Approval No. 2023-SR-646). All data were collected and analyzed according to a prespecified plan fully described in the manuscript. The authors will ensure prospective registration for any future interventional studies.

### 2.6 Intervention measures

All patients received finerenone in combination with guideline-directed medical therapy (GDMT). Patients with HFpEF were treated with finerenone combined with an angiotensin receptor-neprilysin inhibitor (ARNI), a beta-blocker (BB), and a sodium-glucose cotransporter 2 inhibitor (SGLT2i). Patients with non-HFpEF (including HFmrEF and HFrEF) were treated with finerenone combined with ARNI, BB, SGLT2i, and vericiguat, constituting a novel quintuple therapy.

The specific medication administration methods were as follows: Finerenone: Referring to the cardiorenal protection regimen used in studies like FIDELIO-DKD, the starting dose was based on renal function. In this study, the starting dose was 10 mg orally once daily. After 2 weeks of treatment, the dose was individually adjusted based on a comprehensive assessment of the patient’s blood pressure, serum potassium levels, and renal function (e.g., estimated glomerular filtration rate, eGFR). The maximum dose was 20 mg once daily. Sacubitril/Valsartan (ARNI): The starting dose was 50 mg twice daily. The dose was adjusted based on patient tolerance (e.g., blood pressure, serum potassium, renal function, and occurrence of angioedema). After 2 weeks of treatment, if tolerated, the dose could be increased by 50 mg. Thereafter, the dose was assessed and adjusted weekly, with a target dose of 200 mg twice daily or the maximum tolerated dose for long-term maintenance therapy. For patients switching from an angiotensin-converting enzyme inhibitor (ACEI), a 36-h washout period was required before initiating sacubitril/valsartan. For patients switching from an angiotensin II receptor blocker (ARB), a 24-h washout period was required. Beta-Blocker (BB): Metoprolol succinate extended-release tablets (specification: 47.5 mg) were administered orally once daily. Dose titration aimed to control the patient’s resting heart rate between 55 and 70 beats per minute. Dapagliflozin (SGLT2i): A fixed dose of 10 mg was administered orally once daily. Vericiguat: The dose was titrated based on the patient’s blood pressure and clinical symptoms. The starting dose was 5 mg orally once daily. After 2 weeks, if the patient’s systolic blood pressure (SBP) was ≥100 mmHg and well tolerated, the dose could be increased to 10 mg once daily, which was the target maintenance dose. The dose adjustment strategy was as follows: If SBP ≥100 mmHg, consider increasing the dose. If 90 mmHg ≤ SBP <100 mmHg, maintain the current dose. If SBP <90 mmHg, consider reducing the dose if the patient is asymptomatic; interrupt dosing if symptoms occur and restart titration after blood pressure recovers.

#### 2.6.1 Data collection

Data were collected through face-to-face interviews, hospital information systems, and other means. The collected information included: (1) Basic patient information, such as gender, age, body mass index (BMI, defined as weight/height^2^, unit: kg/m^2^), etc.,; (2) Initial laboratory indicators before administration of finerenone combined with GDMT, including white blood cell count (WBC), alanine aminotransferase (ALT), aspartate aminotransferase (AST), total cholesterol (TC), triglyceride (TG), low-density lipoprotein cholesterol (LDL-C), high-density lipoprotein cholesterol (HDL-C), N-terminal pro-B-type natriuretic peptide (NT-proBNP), serum creatinine (Scr), uric acid (UA), urinary albumin-to-creatinine ratio (UACR) levels, etc.,; (3) Clinical assessment results, including the Minnesota Living with Heart Failure Questionnaire (MLHFQ) and the 6-min walking test (6 MW T); (4) All enrolled patients underwent two-dimensional speckle tracking echocardiography (2D-STE) and three-dimensional echocardiography examination (3DE) before enrollment. Echocardiography was re-examined at 1 month and 3 months after starting medication. Laboratory indicators, NT-proBNP, 6 MW T, and MLHFQ were rechecked at 3 months after medication initiation.

The measured cardiac ultrasound data included: Left atrial dimension (LAD), body surface area-based left atrial maximum volume index (LAVi), left atrial ejection fraction (LAEF), left atrial strain during reservoir phase (LASr), left atrial strain during conduit phase (LAScd), left atrial strain during contraction phase (LASct); Left ventricular end-diastolic diameter (LVEDd), left ventricular ejection fraction (LVEF), the ratio of mitral inflow early diastolic velocity to mitral annular early diastolic velocity (E/e'), global longitudinal strain (GLS), cardiac index (CI), left atrioventricular coupling index (LACI); Tricuspid annular plane systolic excursion (TAPSE), right ventricular free wall longitudinal strain (RVFWSL).

#### 2.6.2 STE examination methods and procedures

This study utilized a PHILIPS EPIQ CVX color Doppler ultrasound diagnostic system, equipped with S5-1 and X5-1 phased array probes operating at a frequency range of 2.0–4.5 MHz, and included Auto Strain and Heart Model analysis software. Basic patient information such as name, gender, age, height, and weight was entered into the system prior to analysis.

### 2.7 Electrocardiogram connection

The surface electrocardiogram (ECG) device was connected to display a stable and clear ECG signal, allowing for accurate determination of the patient’s cardiac cycle. The onset of the P-wave on the ECG represents the beginning of atrial contraction, the peak of the R-wave marks the end of ventricular diastole, and the end of the T-wave signifies the end of ventricular contraction. In patients with bundle branch block, the timing of mechanical contraction and cardiac electrical activity may be inconsistent; in such cases, the closing activity of the atrioventricular valves should be used in conjunction to determine the end of ventricular diastole.

### 2.8 Two-dimensional image acquisition

**FIGURE 2:**
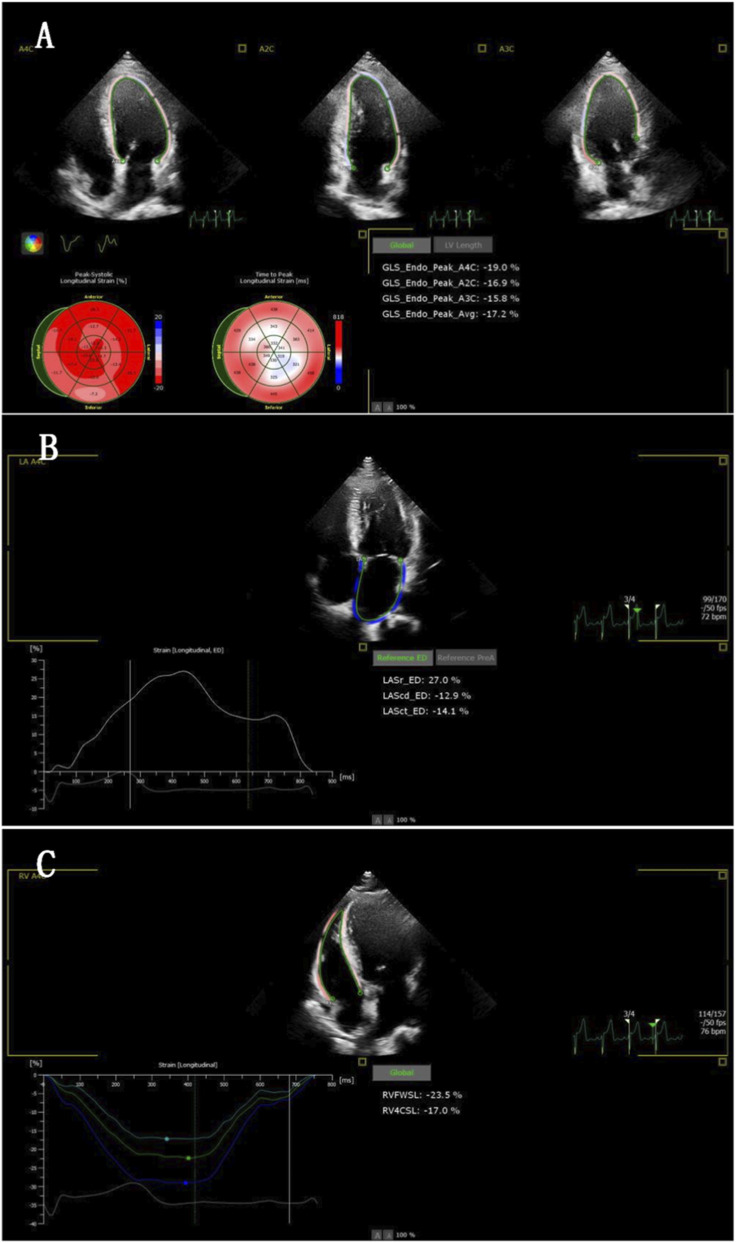
Strain analysis by 2D-STE. **(A)** Measurement of global longitudinal strain (GLS) in the apical 4-chamber, 2-chamber, and 3-chamber views. **(B)** Measurement of left atrial strain (LAS), including reservoir strain (LASr), conduit strain (LAScd), and contractile strain (LASct), in the apical 4-chamber view. **(C)** Measurement of right ventricular free wall longitudinal strain (RVFWSL) in the 4-chamber view.

The patient was placed in the left lateral decubitus position and instructed to maintain quiet breathing. Two-dimensional dynamic image acquisition commenced after the heart rate stabilized. The following key points required attention during image acquisition:

1. Based on the standard apical four-chamber and two-chamber views, parameters such as probe orientation, gain, and depth were finely adjusted to ensure complete visualization of the left atrial structure, including the left atrial free wall, roof (area spanning the pulmonary vein orifices), and interatrial septum.
2. High-quality two-dimensional dynamic images encompassing the apical four-chamber, apical three-chamber (apical long-axis view), and apical two-chamber views were acquired continuously for at least three cardiac cycles to ensure image reproducibility and analytical accuracy.
3. After image acquisition, the analysis interface was accessed. The dedicated strain analysis buttons on the screen were clicked sequentially: “LA Strain” for obtaining left atrial strain parameters, including reservoir strain (LASr), conduit strain (LAScd), and contractile strain (LASct); “LV Strain” for obtaining global longitudinal strain (GLS) of the left ventricle; and “RV Strain” for obtaining right ventricular free wall longitudinal strain (RVFWSL).
4. Multiple measurements were performed for each strain parameter, and the average value was calculated as the final result to enhance data reliability and repeatability. An example of the two-dimensional speckle-tracking echocardiography image acquisition is shown in Figure 2.

### 2.9 Three-dimensional image acquisition

1. Imaging was performed using an X5-1 phased-array transducer with the patient in the left lateral decubitus position.
2. Image optimization for endocardial structure visualization was achieved by adjusting gain, compression, and time-gain compensation controls.
3. 3D datasets were acquired, including wide-angle, single-beat, high-frame-rate (19 ± 3 Hz) 3DE images obtained from the apical window during a single breath-hold.

**FIGURE 3:**
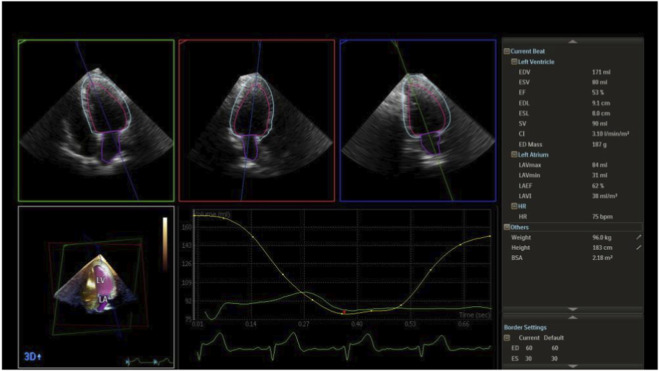
Assessment of left heart function by 3DE.

Endocardial borders of the left ventricular (LV) and left atrial (LA) cavities were automatically traced throughout the cardiac cycle. Manual correction of the automated LV and LA endocardial surfaces was permitted when the operator deemed the automatic detection inaccurate. An example of 3DE image acquisition is shown in Figure 3.

#### 2.9.1 Study endpoints

The primary endpoint was the change from baseline in cardiac structure/function, serum biomarkers, exercise tolerance, and quality of life after treatment. Assessments included: Cardiac structure/function: Assessed via two-dimensional and three-dimensional echocardiography, key parameters included left ventricular ejection fraction (LVEF), global longitudinal strain (GLS), left atrial reservoir strain (LASr), left atrial ejection fraction (LAEF), left atrial conduit strain (LAScd), right ventricular free wall longitudinal strain (RVFWSL), left ventricular end-diastolic diameter (LVEDd), left atrial diameter (LAD), left atrial volume index (LAVi), left ventricular end-diastolic mass index, and left atrioventricular coupling index (LACI). Serum biomarker: NT-proBNP levels. Functional status: 6 MW T distance and MLHFQ score. Follow-up: Patients were assessed at baseline, 1 month, and 3 months after initiating finerenone + GDMT. Quality control: Staff received standardized training. All data were recorded in real-time. Cardiac ultrasound images were independently analyzed by two experienced physicians. Discrepancies were resolved through consensus. Data analysis: Only patients completing all follow-ups were included in the final analysis.

#### 2.9.2 Statistical analysis

This study employed R language (version 4.4.1) for data processing and statistical analysis. All statistical tests were two-sided, and a P-value <0.05 was considered statistically significant. Continuous variables were first assessed for normality using the Shapiro-Wilk test. Variables conforming to or approximately conforming to a normal distribution are described as mean ± standard deviation, while non-normally distributed variables are described as median [P25, P75]. Categorical variables are presented as counts (percentages). Categorical Variables: Comparisons between groups were performed using the Chi-square test or Fisher’s exact test (when more than 20% of cells had an expected count <5). Continuous Variables: Comparison between two groups: The independent samples t-test was used for data with normal distribution and homogeneity of variance; otherwise, the Wilcoxon rank-sum test was used. Comparison among multiple groups: One-way analysis of variance (ANOVA) was used for data with normal distribution and homogeneity of variance, with post-hoc pairwise comparisons conducted using Tukey’s HSD test; otherwise, the Kruskal–Wallis H test was used, with post-hoc pairwise comparisons conducted using Dunn’s test. Within-Group Comparisons and Repeated Measures Data Analysis: Two measurements (e.g., Baseline vs. 3 months): Paired t-tests were used for normally distributed paired data, calculating the mean difference and its 95% confidence interval (CI). Wilcoxon signed-rank tests were used for non-normally distributed paired data, calculating the median difference and its 95% CI. Three or more repeated measurements (e.g., Baseline, 1 month, 3 months): Analyzed using Generalized Estimating Equations (GEE) models to assess the time effect and the time-by-group interaction effect. During model construction, an appropriate working correlation matrix structure (e.g., exchangeable) was selected, and adjustments were made for age, gender, and the baseline level of the corresponding indicator. The model was used to estimate the adjusted mean change from baseline at each time point along with its 95% CI. The between-group difference in treatment effect was assessed by comparing the “difference in changes” between groups.

## 3 Results

### 3.1 Baseline characteristics and medication profile

This study included 67 post-myocardial infarction heart failure patients. The mean age was 57.69 ± 13.98 years, and 71.9% were male. Patients were categorized by LVEF into HFpEF (46.27%), HFmrEF (40.30%), and HFrEF (13.43%). Most patients were in NYHA class II (40.30%) or III (50.75%). Comorbidities included hypertension (34.33%) and diabetes (14.93%). All patients received finerenone combined with guideline-directed medical therapy. ARNI, beta-blockers, and SGLT2 inhibitors were used in 91.04%, 88.06%, and 91.04% of patients, respectively. Vericiguat was administered to 50.75% of patients. Detailed characteristics are shown in Table 1.

**TABLE 1:** Baseline characteristics and medication of patients.

Characteristics	Data	Characteristics	Data
Case (n)	67	Classification of HF according to LVEF	
Gender (male (%))	48 (71.6)	HfpEF [n (%)]	31 (46.27%)
Age (years)	57.69 ± 13.98	HFmrEF [n (%)]	27 (40.30%)
Height (cm)	165.21 ± 6.47	HFrEF [n (%)]	9 (13.43%)
Weight (kg)	70.65 ± 14.26	Medication	
Systolic BP (mmHg)	117.86 ± 14.43	ARNI [n (%)]	61 (91.04%)
Diastolic BP (mmHg)	76.81 ± 7.67	BB [n (%)]	59 (88.06%)
Heart rate (beats/min)	74.78 ± 16.34	Furosemide [n (%)]	31 (46.27%)
Medical history		Finerenone [n (%)]	67 (100%)
Hypertension [n (%)]	23 (34.33%)	SGLT2i [n (%)]	61 (91.04%)
Diabetes mellitus [n (%)]	10 (14.93%)	Vericiguat [n (%)]	34 (50.75%)
Atrial fibrillation [n (%)]	2 (2.98%)	Ezetimibe [n (%)]	40 (59.70%)
NYHA functional class		Statin [n (%)]	61 (91.04%)
I [n (%)]	0 (0%)	Aspirin [n (%)]	54 (80.60%)
II [n (%)]	27 (40.30%)	Clopidogrel [n (%)]	40 (59.70%)
III [n (%)]	34 (50.75%)	Ticagrelor [n (%)]	27 (40.30%)
IV [n (%)]	6 (8.96%)	PCSK9i [n (%)]	36 (53.73%)

### 3.2 Comparison of patient data before and after treatment with finerenone combined with GDMT

Throughout the 3-month follow-up period, all 67 patients survived, with no mortality or recurrent myocardial infarction events reported. After 3 months of combination therapy with finerenone and guideline-directed medical therapy (GDMT), patients demonstrated significant improvements in multiple key parameters. The key cardiac biomarker NT-proBNP decreased significantly from 1,249.34 ± 1,251.17 pg/mL to 431.04 ± 318.92 pg/mL (P < 0.001). Exercise capacity, measured by the 6-min walk test, improved from 381.12 ± 58.64 m to 422.23 ± 49.47 m (P < 0.001), while quality of life, assessed by the MLHFQ score, showed significant improvement with a reduction from 46.81 ± 11.79 to 35.22 ± 8.50 points (P < 0.001).

Renal function parameters also improved, with serum creatinine decreasing from 84.35 ± 20.28 μmol/L to 78.80 ± 15.47 μmol/L (P = 0.006) and urinary albumin-to-creatinine ratio declining from 3.41 ± 1.52 mg/g to 2.76 ± 0.90 mg/g (P < 0.001). A modest reduction in LDL-C was also observed (from 2.71 ± 0.88 mmol/L to 2.34 ± 0.82 mmol/L, P = 0.037).

Safety assessments revealed no significant changes in liver function, electrolytes (including serum potassium), urea, blood glucose, other lipid parameters, or complete blood count during the treatment period (P > 0.05 for all).

These findings indicate that adding finerenone to standard GDMT for 3 months effectively improves cardiac function, renal parameters, exercise tolerance, and quality of life in post-myocardial infarction heart failure patients, with a favorable safety profile. Detailed baseline characteristics and follow-up data are shown in Table 2.

**TABLE 2:** Comparison of biochemical markers and cardiac function parameters in the study population before and after treatment with finerenone combined with GDMT.

Characteristic	Baseline data	3-month follow-up	95% CI	*P*
ALT (U/L)	31.68 ± 13.71	29.97 ± 11.61	−1.71 (−3.99–0.57)	0.137
AST (U/L)	32.07 ± 23.46	27.40 ± 15.65	−4.67 (−14.26–4.92)	0.328
Scr (umol/L)	84.35 ± 20.28	78.80 ± 15.47	−5.55 (−9.39−1.71)	0.006
Urea (mmol/L)	6.58 ± 2.36	6.20 ± 1.72	−0.38 (−1.22–0.46)	0.364
UA (umol/L)	338.18 ± 79.13	325.68 ± 93.50	−12.49 (−43.98–18.99)	0.425
UACR (mg/g)	3.41 ± 1.52	2.76 ± 0.90	−0.65 (−0.92−0.39)	<0.001
TG (mmol/L)	1.48 ± 0.60	1.38 ± 0.57	−0.10 (−0.24–0.05)	0.173
TC (mmol/L)	4.03 ± 1.11	3.80 ± 1.16	−0.23 (−0.67–0.20)	0.285
LDL-C (mmol/L)	2.71 ± 0.88	2.34 ± 0.82	−0.37 (−0.72−0.02)	0.037
HDL-C (mmol/L)	1.26 ± 0.35	1.27 ± 0.32	0.00 (−0.06–0.07)	0.922
LP (a) (mg/L)	246.84 ± 178.20	230.94 ± 141.77	−15.91 (−49.71–17.89)	0.345
GLU (mmol/L)	5.77 ± 1.97	5.83 ± 1.76	0.06 (−0.32–0.44)	0.752
ALB (g/L)	44.43 ± 3.01	45.27 ± 3.39	0.84 (−0.34–2.01)	0.155
K^+^ (mmol/L)	4.08 ± 0.32	4.09 ± 0.34	0.01 (−0.12–0.14)	0.843
Ca^+^ (mmol/L)	2.38 ± 0.11	2.39 ± 0.14	0.01 (−0.03–0.05)	0.630
WBC (10^9^/L)	7.48 ± 2.73	7.00 ± 1.76	−0.48 (−1.14–0.18)	0.151
HGB (g/L)	142.81 ± 17.79	144.38 ± 18.80	1.56 (−2.55–5.67)	0.444
PLT (10^9^/L)	196.25 ± 73.83	192.34 ± 67.04	−3.91 (−20.15–12.34)	0.627
NT-proBNP (pg/mL)	1,249.34 ± 1,251.17	431.04 ± 318.92	−818.30 (−1,212.43−424.18)	<0.001
MWT6 (m)	381.12 ± 58.64	422.23 ± 49.47	41.11 (29.33–52.89)	<0.001
MLHFQ (score)	46.81 ± 11.79	35.22 ± 8.50	−11.59 (−14.02−9.17)	<0.001

Urea, urea carbamide; LP (a), lipoprotein (a); GLU, glucose; ALB, albumin; HGB, hemoglobin; PLT, platelet.

### 3.3 Changes in cardiac structure and function after finerenone combined with GDMT

All 67 patients completed 1- and 3-month follow-up echocardiograms. Generalized estimating equation analysis demonstrated significant improvements in cardiac structure and function parameters following finerenone combined with GDMT therapy.

Cardiac structural parameters showed significant reverse remodeling: left ventricular end-diastolic diameter decreased from (54.85 ± 1.08) mm to (52.85 ± 0.96) mm (change −2.00 mm, P < 0.001); left atrial diameter reduced from (40.62 ± 0.75) mm to (39.56 ± 0.71) mm (change −1.06 mm, P < 0.001); and left atrial volume index declined from (38.78 ± 2.69) mL/m^2^ to (36.25 ± 2.61) mL/m^2^ (change −2.53 mL/m^2^, P < 0.001). Left ventricular mass index and left atrioventricular coupling index also significantly decreased (both P < 0.001).

Cardiac functional parameters substantially improved: left ventricular ejection fraction increased from 49 (2)% to 53 (2)% (change 4%, P < 0.001); global longitudinal strain improved from 14 (1)% to 16 (1)% (change 2%, P < 0.001); left atrial reservoir strain enhanced from 27 (2)% to 32 (2)% (change 6%, P < 0.001); left atrial ejection fraction rose from 47 (3)% to 53 (2)% (change 6%, P < 0.001); and right ventricular free wall longitudinal strain increased from 23 (1)% to 26 (1)% (change 4%, P < 0.001).

**FIGURE 4:**
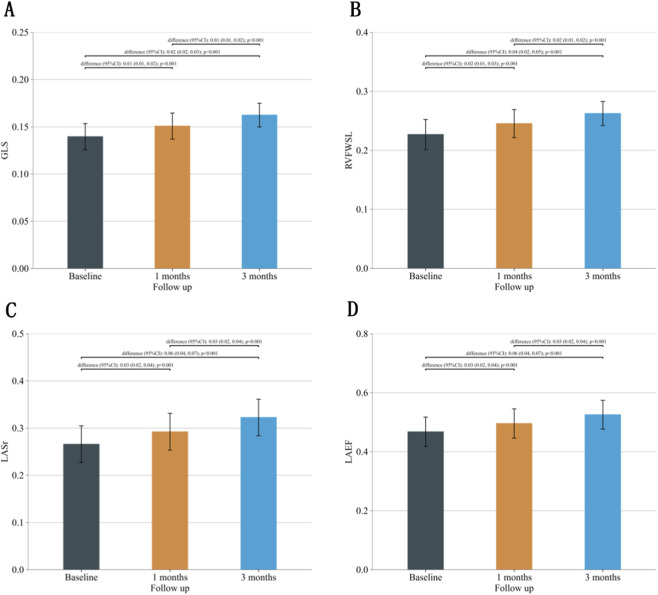
Changes in cardiac function after Finerenone plus GDMT. **(A)** Change in GLS after treatment; **(B)** Change in RVFWSL after treatment; **(C)** Change in LASr after treatment; **(D)** Change in LAEF after treatment.

Improvements exhibited a time-dependent pattern, with most parameters showing significant enhancement at 1 month and further optimization at 3 months (Table 3). Key functional parameters including global longitudinal strain, left atrial reservoir strain, left atrial ejection fraction, and right ventricular free wall longitudinal strain demonstrated sustained upward trends (Figure 4), indicating progressive cardiac functional improvement with extended treatment duration.

**TABLE 3:** Changes in cardiac structure and function following finerenone plus GDMT.

Variable	Baseline	Month 1	Month 3	GEE	Month 1 vs. baseline	Month 3 vs. baseline	Month 3 vs. month 1
LAD (mm)	40.62 (0.75)	40.15 (0.73)	39.56 (0.71)	χ2 = 16.818, *P* < 0.001	−0.47 (−0.71, −0.22); *P* < 0.001	−1.06 (−1.62, −0.50); *P* < 0.001	−0.59 (−1.03, −0.15); *P* = 0.010
LVEDd (mm)	54.85 (1.08)	53.73 (1.01)	52.85 (0.96)	χ2 = 43.891, *P* < 0.001	−1.13 (−1.60, −0.65); *P* < 0.001	−2.00 (−2.63, −1.37); *P* < 0.001	−0.88 (−1.49, −0.26); *P* = 0.006
LVEF (%)	49 (2)	51 (2)	53 (2)	χ2 = 42.095, *P* < 0.001	2 (1, 3); *P* < 0.001	4 (2, 5); *P* < 0.001	2 (1, 3); *P* = 0.004
E/e'	9.93 (0.47)	9.52 (0.47)	9.28 (0.45)	χ2 = 3.725 *P* = 0.155	−0.41 (−1.09, 0.27); *P* = 0.243	−0.64 (−1.31, 0.02); *P* = 0.062	−0.23 (−0.72, 0.25); *P* = 0.342
TAPSE (mm)	20.16 (0.33)	20.88 (0.30)	21.60 (0.26)	χ2 = 39.840, *P* < 0.001	0.72 (0.38, 1.06); *P* < 0.001	1.44 (0.99, 1.89); *P* < 0.001	0.72 (0.34, 1.10); *P* < 0.001
GLS (%)	14 (1)	15 (1)	16 (1)	χ2 = 56.148, *P* < 0.001	1 (1, 2); *P* = 0.001	2 (2, 3); *P* < 0.001	1 (1, 2); *P* < 0.001
LASr (%)	27 (2)	29 (2)	32 (2)	χ2 = 76.237, *P* < 0.001	3 (2, 4); *P* < 0.001	6 (4, 7); *P* < 0.001	3 (2, 4); *P* < 0.001
LAScd (%)	13 (1)	15 (1)	16 (1)	χ2 = 68.792, *P* < 0.001	2 (1, 2); *P* < 0.001	3 (2, 4); *P* < 0.001	2 (1, 2); *P* < 0.001
LASct (%)	13 (1)	14 (1)	16 (1)	χ2 = 34.571, *P* < 0.001	1 (0, 2); *P* = 0.001	2 (2, 3); *P* < 0.001	1 (1, 2); *P* < 0.001
RVFWSL (%)	23 (1)	25 (1)	26 (1)	χ2 = 38.616, *P* < 0.001	2 (1, 3); *P* < 0.001	4 (2, 5); *P* < 0.001	2 (1, 2); *P* < 0.001
ED Mass Index (g/m^2^)	93.20 (3.32)	90.13 (3.16)	88.73 (3.13)	χ2 = 21.251, *P* < 0.001	−3.06 (−4.58, −1.55); *P* < 0.001	−4.47 (−6.37, −2.57); *P* < 0.001	−1.40 (−2.42, −0.39); *P* = 0.008
LAEF (%)	47 (3)	50 (3)	53 (2)	χ2 = 75.220, *P* < 0.001	3 (2, 4); *P* < 0.001	6 (4, 7); *P* < 0.001	3 (2, 4); *P* < 0.001
LAVi (mL/m^2^)	38.78 (2.69)	37.75 (2.68)	36.25 (2.61)	χ2 = 24.981, *P* < 0.001	−1.03 (−1.68, −0.38); *P* = 0.003	−2.53 (−3.56, −1.50); *P* < 0.001	−1.50 (−2.13, −0.87); *P* < 0.001
LACI	0.39 (0.03)	0.37 (0.02)	0.36 (0.02)	χ2 = 25.760, *P* < 0.001	−0.02 (−0.02, −0.01); *P* < 0.001	−0.03 (−0.04, −0.02); *P* < 0.001	−0.01 (−0.02, −0.00); *P* = 0.002

E/e' ratio, an indicator of left ventricular diastolic function; LACI, left atrioventricular coupling index (calculated as LAEDV/LVEDV).

These findings indicate that finerenone combined with GDMT produced significant and progressive improvements in both cardiac structure and function in post-myocardial infarction heart failure patients over 3 months of treatment.

### 3.4 Changes in cardiac function between heart failure subgroups after treatment with finerenone combined with GDMT

To further evaluate the impact of finerenone combined with GDMT on cardiac function in heart failure patients across different left ventricular ejection fraction (LVEF) subgroups, this study divided the 67 patients into an HFpEF group (50% ≤ LVEF <55%, n = 31) and a non-HFpEF group (LVEF <50%, n = 36) based on baseline LVEF. Changes in cardiac structure and function parameters were compared between the two groups before treatment, and after 1 and 3 months of treatment Table 4.

**TABLE 4:** Changes in cardiac function parameters after treatment with finerenone combined with GDMT between HFpEF and non-HFpEF heart failure groups (N = 67).

Variable	Non-HFpEF group (n = 36)	HFpEF group (n = 31)	Changes in the non-HFpEF group	Changes in the HFpEF group	*P*
LAD (mm)					
Before medication	41.46 (0.18)	41.12 (0.11)	-	-	0.191
After 1 month of medication	40.93 (0.21)	40.71 (0.19)	−0.53 (−0.90, −0.17); *P* = 0.005	−0.41 (−0.74, −0.08); *P* = 0.016	0.627
After 3 months of medication	40.19 (0.44)	40.24 (0.28)	−1.27 (−2.29, −0.25); *P* = 0.017	−0.88 (−1.42, −0.34); *P* = 0.002	0.514
LVED (mm)					
Before medication	56.45 (0.17)	55.50 (0.17)	-	-	<0.001
After 1 month of medication	55.25 (0.24)	54.44 (0.33)	−1.20 (−1.79, −0.61); *P* < 0.001	−1.06 (−1.78, −0.34); *P* = 0.005	0.766
After 3 months of medication	53.98 (0.43)	53.91 (0.33)	−2.47 (−3.44, −1.49); *P* < 0.001	−1.59 (−2.35, −0.82); *P* < 0.001	0.165
E/e'					
Before medication	10.03 (0.21)	9.88 (0.16)	-	-	0.615
After 1 month of medication	9.90 (0.45)	9.28 (0.40)	−0.13 (−1.16, 0.90); *P* = 0.800	−0.61 (−1.51, 0.30); *P* = 0.192	0.499
After 3 months of medication	9.04 (0.51)	9.48 (0.40)	−0.99 (−1.98, −0.01); *P* = 0.052	−0.40 (−1.28, 0.48); *P* = 0.377	0.380
TAPSE (mm)					
Before medication	19.92 (0.13)	20.14 (0.11)	-	-	0.232
After 1 month of medication	20.52 (0.20)	20.96 (0.21)	0.60 (0.08, 1.12); *P* = 0.025	0.82 (0.38, 1.26); *P* < 0.001	0.518
After 3 months of medication	21.39 (0.31)	21.55 (0.22)	1.47 (0.71, 2.23); *P* < 0.001	1.41 (0.89, 1.93); *P* < 0.001	0.907
GLS (%)					
Before medication	13 (0.00)	14 (0.00)	-	-	0.011
After 1 month of medication	15 (0.00)	15 (0.00)	1 (0, 3); *P* = 0.021	1 (0, 1); *P* = 0.001	0.410
After 3 months of medication	16 (0.00)	16 (0.00)	3 (2, 4); *P* < 0.001	2 (1, 2); *P* < 0.001	0.081
LASr (%)					
Before medication	24 (0.00)	25 (0.00)	-	-	0.065
After 1 month of medication	27 (0.01)	28 (0.01)	3 (1, 4); *P* = 0.001	2 (2, 3); *P* < 0.001	0.661
After 3 months of medication	31 (0.01)	30 (0.01)	6 (4, 8); *P* < 0.001	5 (4, 6); *P* < 0.001	0.330
LAScd (%)					
Before medication	12 (0.00)	13 (0.00)	-	-	0.030
After 1 month of medication	14 (0.00)	14 (0.00)	2 (1, 3); *P* < 0.001	1 (1, 2); *P* < 0.001	0.216
After 3 months of medication	16 (0.01)	16 (0.00)	4 (3, 5); *P* < 0.001	3 (2, 3); *P* < 0.001	0.117
LASct (%)					
Before medication	12 (0.00)	12 (0.00)	-	-	0.377
After 1 month of medication	13 (0.01)	13 (0.00)	1 (−0, 2); *P* = 0.086	1 (1, 1); *P* < 0.001	0.985
After 3 months of medication	15 (0.01)	15 (0.00)	3 (1, 4); *P* < 0.001	2 (1, 3); *P* < 0.001	0.942
RVFWSL (%)					
Before medication	21 (0.00)	23 (0.00)	-	-	0.009
After 1 month of medication	23 (0.01)	25 (0.01)	2 (1, 3); *P* = 0.002	2 (1, 3); *P* = 0.004	0.633
After 3 months of medication	25 (0.01)	0.26 (0.01)	4 (2, 5); *P* < 0.001	3 (2, 5); *P* < 0.001	0.917
ED Mass Index (g/m^2^)					
Before medication	95.81 (0.80)	93.32 (0.56)	-	-	0.051
After 1 month of medication	91.75 (1.01)	91.12 (0.79)	−4.05 (−6.88, −1.23); *P* = 0.006	−2.19 (−3.42, −0.96); *P* = 0.001	0.237
After 3 months of medication	90.01 (1.25)	90.02 (1.04)	−5.79 (−9.18, −2.41); *P* = 0.001	−3.30 (−5.09, −1.51); *P* = 0.001	0.202
LAEF (%)					
Before medication	45 (0.01)	45 (0.00)	-	-	0.686
After 1 month of medication	47 (0.01)	48 (0.01)	3 (1, 4); *P* < 0.001	3 (2, 4); *P* < 0.001	0.682
After 3 months of medication	51 (0.01)	0.50 (0.01)	6 (5, 8); *P* < 0.001	5 (3, 7); *P* < 0.001	0.324
LAVi (mL/m^2^)					
Before medication	39.00 (0.20)	38.78 (0.10)	-	-	0.417
After 1 month of medication	37.60 (0.55)	38.08 (0.25)	−1.40 (−2.65, −0.15); *P* = 0.031	−0.71 (−1.19, −0.22); *P* = 0.005	0.310
After 3 months of medication	36.33 (0.89)	36.37 (0.48)	−2.67 (−4.58, −0.76); *P* = 0.007	−2.41 (−3.38, −1.45); *P* < 0.001	0.815
LACI					
Before medication	0.41 (0.00)	0.40 (0.00)	-	-	0.309
After 1 month of medication	0.39 (0.00)	0.39 (0.00)	−0.02 (−0.02, −0.01); *P* = 0.001	−0.02 (−0.03, −0.01); *P* = 0.002	0.932
After 3 months of medication	0.38 (0.01)	0.38 (0.01)	−0.03 (−0.04, −0.01); *P* = 0.006	−0.03 (−0.04, −0.01); *P* < 0.001	0.894

Footnote: Data are presented as mean (SE) or median [IQR].

The results showed that both groups demonstrated significant improvements in cardiac structure and function after treatment. Regarding cardiac structure, parameters including left atrial diameter (LAD), left ventricular end-diastolic diameter (LVEDd), end-diastolic mass index (ED Mass Index), left atrial volume index (LAVi), and left atrioventricular coupling index (LACI) were significantly reduced from baseline in both groups (all intra-group comparisons at all time points, P < 0.05), suggesting that finerenone combined with GDMT promotes cardiac reverse remodeling. Regarding cardiac function, parameters including left ventricular ejection fraction (LVEF), global longitudinal strain (GLS), left atrial reservoir strain (LASr), left atrial ejection fraction (LAEF), tricuspid annular plane systolic excursion (TAPSE), and right ventricular free wall longitudinal strain (RVFWSL) were significantly improved from baseline in both groups (all intra-group comparisons at all time points, P < 0.05), indicating comprehensive improvement in left ventricular systolic function, left atrial function, and right ventricular function.

**FIGURE 5:**
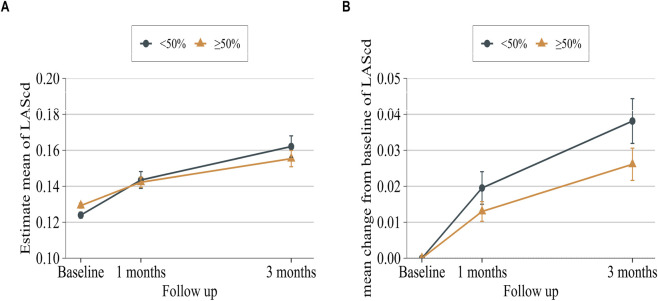
Changes in LAScd in the two patient groups after treatment with finerenone combined with GDMT. **(A)** Mean changes in LAEF at baseline, 1 month, and 3 months of treatment. **(B)** Mean changes in LAEF at 1 month and 3 months compared to baseline (pre-treatment). As shown, the green line represents the non-HFpEF group, and the yellow line represents the HFpEF group.

**FIGURE 6:**
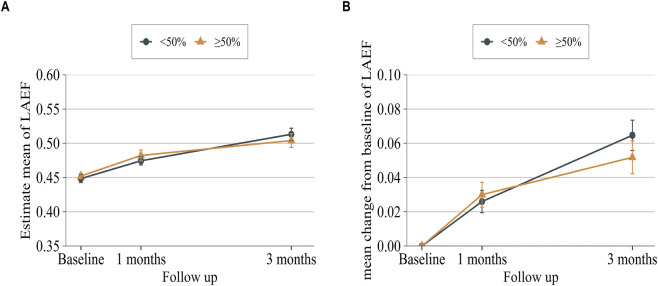
Changes in LAEF in the two patient groups after treatment with finerenone combined with GDMT. **(A)** Mean values of LAScd at 1 month and 3 months of treatment. **(B)** Mean changes in LAScd at 1 month and 3 months compared to baseline (pre-treatment). As shown, the green line represents the non-HFpEF group, and the yellow line represents the HFpEF group.

Notably, both left atrial conduit strain (LAScd) and LAEF showed continuous and significant improvement over time in both groups (P < 0.001 at all-time points). Inter-group comparisons revealed that the non-HFpEF group showed a numerically greater magnitude of improvement in LAScd and LAEF compared to the HFpEF group (Figures 5, 6). However, this trend did not reach statistical significance (interaction P > 0.05 for all comparisons). Therefore, the observed numerical differences should not be overinterpreted.

In conclusion, finerenone combined with GDMT significantly improved cardiac structure and function in both HFpEF and non-HFpEF patients over 3 months, with particularly notable benefits on left atrial function.

## 4 Discussion

This prospective real-world study of 67 patients with post-myocardial infarction heart failure (including both HFpEF and non-HFpEF) over 3 months preliminarily investigated the short-term effects of combining finerenone with guideline-directed medical therapy (GDMT) on cardiac function, cardiac structure, serum biomarkers, exercise tolerance, and quality of life. The results showed that the finerenone plus GDMT regimen provided significant benefits in improving left ventricular systolic and diastolic function, reversing cardiac remodeling, reducing N-terminal pro-B-type natriuretic peptide (NT-proBNP) levels, and enhancing exercise tolerance and quality of life. Furthermore, the combination therapy was associated with a significant reduction in UACR, suggesting a potential early renoprotective signal that requires further investigation, with a favorable safety profile and no significant hyperkalemia or renal function deterioration.

In recent years, the dual role of mineralocorticoid receptor (MR) overactivation in the progression of both heart failure and renal injury has gained increasing attention. Finerenone, as a novel non-steroidal MR antagonist (MRA), possesses higher receptor selectivity and affinity (Kolkhof et al., 2021). The phase III FINEARTS-HF trial confirmed that in symptomatic heart failure patients with a left ventricular ejection fraction (LVEF)≥40%, finerenone significantly reduced the risk of the composite endpoint of cardiovascular death and heart failure events by 16%, with benefits emerging as early as 28 days after initiation (Solomon et al., 2024b). The results of our study are highly consistent with the main findings of FINEARTS-HF (Solomon et al., 2024a; Docherty et al., 2025), further validating the early cardiorenal protective potential of finerenone in a broad population encompassing both heart failure with mildly reduced ejection fraction (HFmrEF) and HFpEF in a real-world setting.

From a mechanistic perspective, the cardiorenal protective effects of finerenone may stem from its precise modulation of the MR signaling pathway. MR overactivation promotes the expression of various pro-fibrotic and pro-inflammatory factors (such as TGF-β, PAI-1, IL-6, etc.), leading to cardiomyocyte hypertrophy, interstitial fibrosis, vascular endothelial dysfunction, and renal podocyte injury (El Mouhayyar et al., 2025; Bauersachs and Lopez-Andres, 2022). As a highly selective MRA, finerenone not only effectively antagonizes aldosterone-mediated MR activation but also demonstrates more balanced distribution at the tissue level between the heart and kidneys, thereby more effectively inhibiting organ fibrosis and inflammatory responses (Agarwal et al., 2021). Recent basic research further suggests that finerenone may exert synergistic cardiorenal protective effects through multiple mechanisms, including modulating macrophage polarization, improving mitochondrial function, and alleviating oxidative stress (Zhai et al., 2024; Kolkhof et al., 2014).

Notably, in this study, after combination therapy with finerenone, patients showed significant improvements not only in cardiac function parameters such as LVEF, global longitudinal strain (GLS), left atrial reservoir strain (LASr), and left atrial ejection fraction (LAEF), but also marked reversal in cardiac remodeling indicators such as left atrial diameter (LAD), left ventricular end-diastolic diameter (LVEDd), and left atrial volume index (LAVi). This suggests that finerenone may delay or even partially reverse adverse ventricular and atrial remodeling by inhibiting myocardial fibrosis and inflammatory responses. The particularly significant improvement in left atrial function parameters (e.g., LASr, LAEF) indicates a positive impact on left atrial function, which is less commonly observed in previous MRA studies and might be related to its stronger anti-fibrotic mechanism and its regulatory effect on the atrium-specific electromechanical environment. Furthermore, the significant reduction in the urinary albumin-to-creatinine ratio (UACR) further suggests that finerenone exerts early anti-inflammatory and anti-fibrotic effects at the renal level. This echoes observations from the FIDELIO-DKD and FIGARO-DKD studies, where finerenone significantly delayed chronic kidney disease progression and reduced albuminuria (Agarwal et al., 2022; Sato and Nishimoto, 2025), indicating its potential for early renal protection also in post-myocardial infarction heart failure patients.

Additionally, this study found that the trends in structural and functional improvements brought by finerenone were largely consistent, with no significant differences between groups, regardless of whether patients belonged to the HFpEF or non-HFpEF category based on baseline LVEF. All interaction P-values were >0.05, confirming the absence of a statistically significant differential treatment effect. This finding resonates with the consistent efficacy of finerenone across the LVEF spectrum observed in the FINEARTS-HF subgroup analysis (Vaduganathan et al., 2024; Docherty et al., 2025), further supporting its broad application potential in heart failure patients across the full ejection fraction spectrum, especially in the HFpEF population where current treatment options are relatively limited. In recent years, the international academic community has increasingly emphasized the role of the MR pathway in the pathogenesis of HFpEF, particularly in patients with comorbidities like metabolic syndrome and diabetes, where MR overactivation may exacerbate diastolic dysfunction by promoting microvascular inflammation and increasing myocardial stiffness (Savarese et al., 2024; Pandey et al., 2022). Due to the tissue penetration and safety advantages conferred by its non-steroidal structure, finerenone holds promise as a new cornerstone for HFpEF treatment.

Regarding safety, in this study, patients’ serum potassium and renal function indicators remained stable during the 3-month follow-up period, consistent with the results from the FINEARTS-HF study, which reported a low incidence of hyperkalemia in the finerenone group and an overall safety profile comparable to placebo (Vaduganathan et al., 2024). Although in the FIDELIO-DKD study (Bakris et al., 2020), the incidence of hyperkalemia-related adverse events was higher with finerenone compared to placebo (18.3% vs. 9.0%), the proportion of discontinuations due to hyperkalemia remained low (2.3% vs. 0.9%), and no hyperkalemia-related deaths were reported. This study further corroborates the good tolerability and manageable safety profile of finerenone in real-world clinical practice, suggesting high clinical application safety in the Chinese population under standardized monitoring.

In conclusion, this real-world study of 67 patients preliminarily confirms that in patients with acute post-myocardial infarction heart failure, the addition of finerenone to GDMT can significantly improve cardiac structure and function, reduce NT-proBNP levels, enhance exercise capacity and quality of life, and is associated with a significant reduction in UACR, all with a favorable safety profile over the short term. Given the single-arm design and moderate sample size, these findings are exploratory and hypothesis-generating. This provides an academic basis for designing future randomized controlled trials to confirm the cardiorenal benefits of finerenone in this specific population.

## Data Availability

The original contributions presented in the study are included in the article/supplementary material, further inquiries can be directed to the corresponding author.
